# Quantitative and Fast Sterility Assurance Testing of Surfaces by Enumeration of Germinable Endospores

**DOI:** 10.1038/s41598-019-57175-3

**Published:** 2020-01-16

**Authors:** Pun To Yung, Elizabeth Lester, Adrian Ponce

**Affiliations:** 1 Jet Propulsion Laboratory, California Institute of Technology, Pasadena, CA USA; 2 Present Address: Department of Biomedical and Chemical Engineering, Syracuse University, Syracuse, NY USA; 3 Present Address: Verrix, LLC, San Clemente, CA USA

**Keywords:** Assay systems, Applied microbiology

## Abstract

A fast Endospore Germinability Assay (EGA) was validated with traditional plate counts to enumerate single endospore germination events for monitoring surface sterilization. The assay is based on a time-gated luminescence microscopy technique enabling visualization and enumeration of individual germinating endospores. Germinating endospores release calcium dipicolinate to form highly luminescent terbium dipicolinate complexes surrounding each germinating endospore. EGA and heterotrophic plate counting (HPC) were used to evaluate the swab/rinse recovery efficiency of endospores from stainless steel surfaces. EGA and HPC results were highly correlated for endospore recovery from stainless steel coupons inoculated with range of 1,000 endospores per coupon down to sterility. Dosage-dependent decrease of surface endospore germinability were observed in dry heat, UV irradiation, oxygen plasma and vaporized hydrogen peroxide treatments, measured with EGA and HPC. EGA is a fast and complementary method to traditional HPC for quantitative sterility assurance testing of surfaces. This work introduces and validates a 15-minute or faster assay for germinable endospores to complement the conventional lengthy, culture-based surface sterility validation, which is critical in hospitals, food and pharmaceutical industries to help minimize nosocomial infection, food spoilage, and pharmaceutical contamination.

## Introduction

Even before the era of microbiology, people adopted cooking, bathing and washing garments to reduce the devastating effects of disease caused by infectious pathogens^1,2^. Cleansing and purification of materials have been employed to improve human health since Roman times, but it was not until the 1800s when Louis Pasteur and William Henry explored pressurized steam for sterilization of contaminated clothing and materials that have been in contact with contagions^3^. The ongoing co-evolution of pathogen and human has been described as an arms race, where new developments of antimicrobial medicines are overcome by microbes with newly evolved resistance to those drugs^4–7^. Indeed, as drug development fails to keep pace with increased microbial resistance, hygiene and monitoring of sterility assurance are becoming increasingly important.

Because of the persistence of nosocomial infections and foodborne diseases, much attention in health care, food and pharmaceutical industries has focused on the need for appropriate surface cleaning, disinfection, and sterility assurance. The cleanliness of surfaces, such as walls, countertops, and floors, in hospitals have been shown to correlate with the occurrence of cross-contamination^8–11^. In the food industry, surfaces in contact with food are critical, because food provides nutrients and substrate for microbes to proliferate. Inadequate cleaning and disinfection of these surfaces put consumers at risk, and leads to food spoilage or shortened shelf lives^12,13^. Quality assurance methods in the pharmaceutical industry to prevent microbial contamination are also strictly regulated to ensure aseptic processing of products^14,15^.

Sterility assurance is also important in space exploration, where nations are required by international law to ensure that interplanetary spacecraft destined to potentially habitable worlds in our solar system contain less than a specified bioburden^16,17^. For example, Mars lander missions without life-detection experiments must meet a bioburden limit of 3 × 10^5^ endospores/vehicle, and less than 300 endospores/m^2^. Missions with life-detection experiments must undergo additional procedures to ensure that the total bioburden is even lower^18^. The standards pertaining to bioburden use endospores (i.e., bacterial spores) as the indicator organisms to verify that bioburden thresholds are not exceeded. Endospores are ideal bioindicator organisms because they are the most resistant form of life towards various sterilization regimens, such as dry heat, steam, vaporized hydrogen peroxide, ethylene oxide, oxygen plasma and ultraviolet radiation^19–23^.

Evaluation of microbial contamination on work surfaces has relied on the swab and rinse sampling techniques and standard heterotrophic plate count (HPC), which have been widely practiced in hospitals, food processing plants, and by NASA for evaluating the bioburden level of flight hardware for planetary protection in robotic extraterrestrial missions^24,25^. The major drawback is that HPC usually requires several days for results. Faster and more efficient methods have been proposed in recent years to measure microbial contamination of surfaces, such as ATP bioluminescence, enzyme, and nucleic acid-based techniques^26–30^. Yet, conventional cleaning practices of surfaces still largely do not include quantitative approaches for assessing the effectiveness of the cleaning regimen, because of cost, ease-of-use, or time-to-results. There is a need for fast, quantitative, cost effective and convenient methods to assess the level of contamination on surfaces in tracking routes of infection, surveillance of the environment and evaluation of decontamination protocols. The endospore germinability assay (EGA) addresses this need by enabling the fast measurement of germinable endospore populations, which serve as excellent indicators for measuring surface bioburden reduction, owing to their omnipresence in the environment, and because they are the hardiest microbial organisms. Consequently, a reduction of viable endospores necessarily indicates an even greater reduction in non-spore forming microorganisms.

The concept of endospores as bioindicators for monitoring sterilization effectiveness is extensively used in hospitals, where sterilization processes are monitored by using endospores as dosimeters (e.g., endospore test strips to verify autoclave compliance with regulatory requirements^31^). Endospore producing genera include *Geobacillus*, *Bacillus* and *Clostridium*, which produce this dormant and resilient form during times of environmental stress. They are protected from environmental extremes and sterilants by a series of protective layers, including spore coat, peptidoglycan cortex, and a core that includes high concentrations of calcium dipicolinate stores^32–34^. Endospores can remain dormant for many years^35,36^, and when more favorable conditions are signaled by the presence of water, nutrients, and germinants, endospores may germinate and become metabolically active^22,37,38^. In the context of human health, this enables endospore-forming pathogens (e.g., *B*. *anthracis*, *C*. *botulinum, and C. difficile*) to persist after cleaning regimens are applied, causing contamination, food spoilage, and disease. The remarkable resistance of endospores makes them ideal bioindicators for both sterility assurance of sterilizers and of surfaces in work environments of health care, food and pharmaceutical industries, and for planetary protection as humans continue to explore potentially habitable worlds in the solar systems.

We have previously reported on germinable-endospore biodosimetry using EGA to achieve rapid validation of sterilization in aqueous suspensions^39^. DPA (dipicolinic acid, 2,6-pyridinedicarboxylic acid) naturally present in endospores can be released from the core by inducing germination (e.g., with L-alanine^40,41^) or physical lysis (e.g., autoclaving, microwaving^42^). L-alanine is a general germinant for endospores, and the addition induces endospores to germinate and release approximately 10^8^ DPA molecules per endospore. When endospores germinate and release their Ca-DPA stores into the immediate surrounding volume, the resultant Tb-DPA luminescent complex surrounding the germinated endospore bodies,  are imaged with a time-gated microscope. DPA acts as a light-harvesting antenna with a large extinction coefficient in the UV to transfer the energy to Tb^3+^ upon UV excitation^43–46^. An intensity profile can be recorded in accordance with the lag time and germination dynamics on a time scale of 15 minutes for most germinable spores in a population. Only a small subset of the germinable population germinates > 15 minutes, even for populations that have been exposed to inactivation regimens. Each Tb-DPA luminescent spot in the time-gated microscope field of view is assigned and enumerated as one germinable endospore.

In this paper, we focus on the application of EGA for detecting germinable endospores on test surfaces, which involves a 4-step procedure consisting of (i) swab-rinse sampling, (ii) sample preparation, (iii) time-gated imaging with EGA, and (iv) enumeration of germinable endospores^39,47^. EGA has also been used to investigate germinable endospore populations within environmental samples, including ice cores^39,48^, desert soils^39,48^, and an Antarctic lake^49^, and has been adapted for *Clostridium* endospores^48,50^. The objectives of this investigation are to (1) validate the EGA methodology in comparison to standard HPC to assess the number of endospores sampled from nonporous stainless steel coupon surfaces, and (2) employ EGA in comparison to HPC for determination of the decimal reduction values of surface endospore populations inactivated by dry heat, ultraviolet radiation, vaporized hydrogen peroxide, and atmospheric pressure air plasma.

## Results and Discussion

### Germination and culturing as a measure of endospore viability

The Endospore Germinability Assay (EGA) is based on the earliest observable mechanistic steps of dormant endospores reentering the active life cycle as observed by DPA release during stage I germination. By comparison, ATP assays probe at stage II germination and culturing probes after 20 or so replication cycles. None of these methods directly probe the viable population. Defining viability for microorganisms is neither simple nor straightforward^51^. Endospores that give rise to colonies on growth media are clearly viable, and this is generally considered a reliable approach to demonstrating viability. However, culture-based methods often overlook more than 99% of the viable-but-not-culturable (VBNC) population of cells in environmental samples^52–54^, effectively representing a conservative lower limit estimate of viability. In contrast, germination accounts for a greater fraction of viable organisms, but may include a subset population of germinable-but-not-viable endospores. As a complement to culturability, however, germination may be considered an upper limit on the viable number of endospores. While germination is a process that is nutrient-induced, and nutrients supplied may not cause all spores to germinate^55,56^, we have demonstrated the reproducible correlation of germination-capable populations to culturable populations within lab spore suspensions, and that this correlation can be extended to spore inactivation studies^39,57^. Therefore, trends observed in germinability can be used as a good indication of trends in viability, along with assigning an upper limit to the viable population.

Quantification of all viable biomass, as defined as having the ability for metabolic activity and reproduction, in an environmental sample, is currently difficult to achieve. Nonetheless, the culture-based assays and the Endospore Germinability Assay may be used in parallel to map out upper and lower limits of viability, thus allowing a bounded estimate of viability.

The application of EGA for sterility assurance of surfaces may be implemented by measuring the reduction in germinable endospore populations before versus after surface sterilization regimen are applied, which provides a measure of sterility assurance level. While endospore presence on surfaces are generally ubiquitous, a mixed population of environmental endospores may include species that are not readily germinable on the EGA experiment time scale with the germinants supplied. This concern may be mitigated by a broad spectrum germinant solution and extending the EGA observation time. Alternatively, fully characterized, bioindicator endospore populations may be used on surfaces to assess sterilization effectiveness with EGA. Additional characterization of EGA applied to surface sterility assurance with various sterilants and environments will provide improved guidelines for appropriate use of EGA for a range of applications.

### Recovery of endospores from test surface coupons of stainless steel

**Figure 1:**
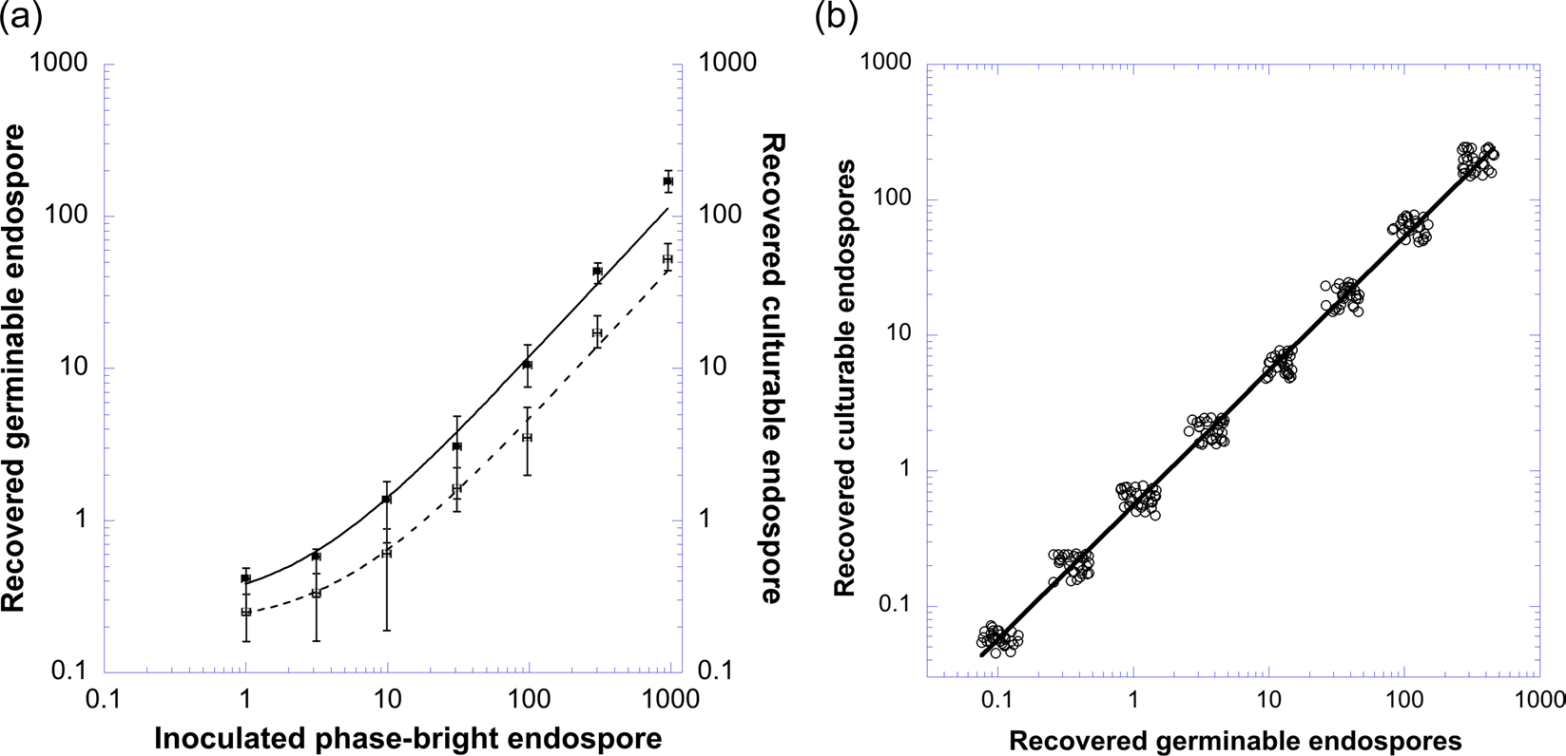
Recovery of endospores on stainless steel coupon surface using EGA (solid square, solid line, *n* = 180) and TSA HPC (open square, dashed line, *n* = 240) on a log-log plot. (**a**) Recovered germinable endospores were plotted against inoculated germinable endospores; recovered culturable endospores were plotted against inoculated culturable endospores. (**b**) Recovered germinable and culturable endospores were both plotted against inoculated phase-bright endospores. Error bars indicate the standard deviation on quintuplicate measurements.

Coupon surfaces were inoculated with a range of total spore populations as determined by phase contrast microscopy (0, 0.3, 1, 3.2, 10, 31.6, 100, 317, 1000 spores/coupon), of which 48% were germinable and 28% were culturable. The coupon surfaces were swabbed, rinsed and assayed by both EGA and HPC. Endospore recovery percentages (%R) were determined to be 36.3 ± 7.6% for EGA (%R_EGA_) and 19.8 ± 7.2% for HPC (%R_HPC_) (Fig. 1a). EGA and HPC results were strongly correlated as determined by Spearman rank correlation coefficient (*r*_*s*_ = 0.9321, *p* < 0.0001) on the entire data set. A scatter plot of EGA-HPC results and pairwise regression analyses (Fig. 1b) revealed a correlation coefficient of 0.965 across almost four orders of magnitude in concentration, as well as a strong linear correlation. The limit of detection (LOD) for endospore detection by EGA was calculated to be single endospores based on the fact that all signals had a signal-to-noise ratio (S/N) greater than or equal to 3. No false positives were recorded by either assay, as all sterile coupons gave zero counts.

The swab-and-rinse procedure consists of two major steps, where Step 1 is the transfer of endospores from the coupon surface to the swab head, and Step 2 is the transfer of endospores from the swab head into aqueous suspension by a series of vortexing and sonication. While step 1 is fundamentally difficult to assess empirically, we carried out an experiment on the recovery of endospores directly inoculated onto the cotton tip of a swab applicator. The inoculum population was 1,000 ± 67.5 endospores (*n* = 20, of which 48% were germinable and 28% were culturable). Table 1 shows that the recovery efficacies measured by EGA was 92.7 ± 9.2% and TSA HPC was 86.8 ± 13.8%.

**Table 1:** Percentage of endospores recovered from swabs directly inoculated with 1000 ± 67.5 endospores (*n* = 20, of which 48% were germinable and 28% were culturable). Percentages calculated are relative to mean of control tests, thus allowing maximum to be > 100%. CI: confidence interval.

	Mean	Median	SD	Range	95% CI
EGA	92.7	93.0	9.2	75.0–107.7	87.5–98.5
TSA pour plating	86.8	84.5	13.8	64.0–114.3	73.9–103.5

EGA values were calculated based on recovered germinable endospores against inoculated germinable endospores. TSA pour plating values were calculated based on recovered culturable endospores against inoculated culturable endospores.

### Thermal inactivation of *B. atrophaeus* endospores

**Figure 2:**
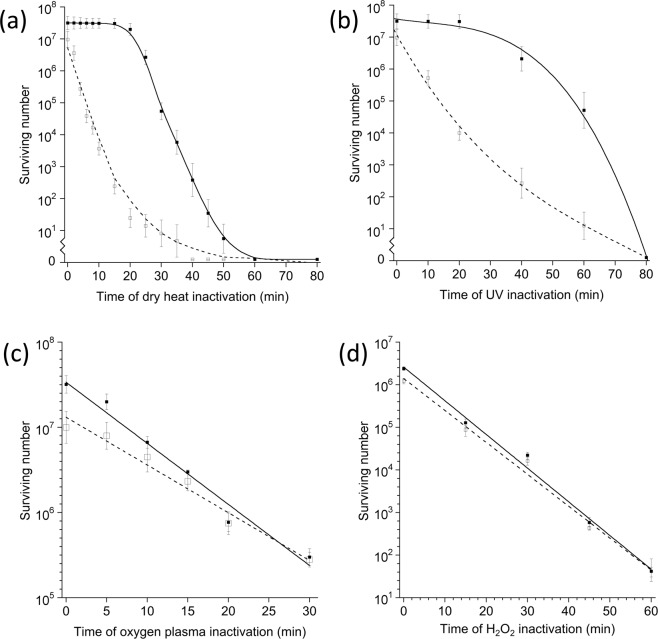
Inactivation of *B*. *atrophaeus* endospores inoculated on stainless steel coupons showing EGA (solid line) and heterotrophic plate (dashed line) counts under **(a)** 105 °C dry heat inactivation. **(b)** UV inactivation with a mercury lamp irradiating samples at 254 nm with a power of 22.9 µW/cm^2^ on endospores on surface. **(c)** Inactivation of *B*. *subtilis* endospores inoculated on PDMS as a function of oxygen plasma inactivation time. **(d)** Vaporized hydrogen peroxide inactivation of *G*. *stearothermophilus* endospores from spore strips.

Figure 2a shows semi-logarithmic survival graphs of *B*. *atrophaeus* endospores laden on stainless steel coupons subjected to dry heat inactivation at 105 °C as measured by EGA and HPC. Survival graphs showed significant reduction of viable endospore counts with increasing thermal treatment time. The coupon was rendered sterile as measured by both EGA and HPC after 60 minutes at 105 °C (>7 log reduction). Survival graphs measured were fitted to both log-linear and Weibull distribution models. The kinetics and goodness-of-fit parameters are shown in Table 2. The survival graph measured by EGA showed a sigmoid curve with a noticeable 18-min shoulder and tail, while the HPC graph showed log linear decay of endospore viability. To verify the existence of shoulder on the EGA graph, 6 data points were taken within the initial 10 min. The EGA *D*-value was calculated based on the log-linear portion of the EGA sigmoidal inactivation graph, which yielded a *D*-value of 25.6 ± 3.2 min and *t*_*C*_ of 14.7 ± 2.1 min. The HPC inactivation graph yielded a *D*-value of 3.4 ± 0.6 min and *t*_*C*_ of 2.64 ± 0.3 min. The HPC inactivation graph measured showed upward concavity, which indicates that at least two populations of endospores were present, with one that was inactivated at a relatively fast rate leaving behind survivors with a higher resistance.

**Table 2:** Kinetic parameters of log-linear and Weibull models for *B*. *atrophaeus* endospores subjected under thermal inactivation and ultraviolet inactivation on surfaces, *B*. *subtilis* endospores subjected under oxygen plasma inactivation, and *G*. *stearothermophilus* endospores challenged with vaporized hydrogen peroxide. All the time parameters were displayed in min. All data were represented as means ± standard deviation of the mean. The mean data were analyzed by Student’s *t* test. The level of significance was considered at *p* < 0.05. Goodness-of-fit of the models was validated by *R*^2^ values.

Inactivation	EGA						Culture					
	Linear		Weibull				Linear		Weibull			
	*D*	*R*^2^	*α*	*β*	*t*_*c*_	*R*^2^	*D*	*R*^2^	*α*	*β*	*t*_*c*_	*R*^2^
Dry heat	25.6 ± 3.2	0.93	15.4 ± 1.7	1.55 ± 0.2	14.7 ± 2.1	0.97	3.4 ± 0.6	0.95	2.8 ± 0.2	0.65 ± 0.1	2.6 ± 0.3	0.98
Dry UV	12.4 ± 4.5	0.80	7.7 ± 0.4	2.29 ± 0.5	6.9 ± 0.5	0.97	11.6 ± 3.8	0.90	6.8 ± 0.5	0.88 ± 0.1	6.1 ± 0.3	0.9
O_2_ plasma	14.0 ± 1.6	0.99	14.4 ± 1.0	1.02 ± 0.1	14.2 ± 1.2	0.99	16.9 ± 4.0	0.99	17.5 ± 2.2	1.01 ± 0.1	17.2 ± 2.3	0.99

### Ultraviolet inactivation of *B. atrophaeus* endospores

Figure 2b shows the inactivation graphs of *B*. *atrophaeus* endospores air-dried on stainless steel coupons, subjected to 254-nm irradiation with a power of 22.9 µW/cm^2^. EGA and HPC were employed to enumerate the surviving endospore fractions. Survival graphs were fitted to both the log-linear and Weibull models. EGA measured an inactivation curve with downward concavity, while HPC generated an inactivation curve that followed a log-linear pattern with a slight upward concavity. The EGA and HPC inactivation curves show substantial differences, which indicates that germination stage Imechanisms are less susceptible to UV inactivation than replication mechanisms. Sterility was achieved after 80 min of ultraviolet irradiation as measured by both assays. EGA measured a *D*-value of 12.4 ± 4.5 min and *t*_*C*_ of 6.9 ± 0.5 min. HPC measured a *D*-value of 11.6 ± 3.8 min and *t*_*C*_ of 6.1 ± 0.3 min.

### Oxygen plasma inactivation of *B. subtilis* endospores

Figure 2c shows survival graphs of *B*. *subtilis* endospores inactivated by oxygen plasma. EGA and HPC both yielded first-order exponential inactivation kinetics from 0 to 30 min with good fitting to both log-linear and Weibull distribution plots. A rapid loss in germinability and culturability was observed after 30 min of exposure. The two methods were highly correlated with a correlation coefficient of 0.9997. EGA measured a *D*-value of 14.0 ± 1.6 min and *t*_*C*_ of 14.2 ± 1.2 min. HPC measured a *D*-value of 16.9 ± 4.0 min and *t*_*C*_ of 17.2 ± 2.3 min. The survival curve showed a significant reduction of the viable endospore count for increasing plasma inactivation time. The Weibull shape parameters, *β*, were very close to 1 in both cases because of the log-linear shape of the inactivation graphs.

### Vaporized hydrogen peroxide inactivation of *G. stearothermophilus* endospores

Figure 2d illustrates the inactivation data of *G*. *stearothermophilus* endospores as a function of vaporized hydrogen peroxide treatment time. The data were fitted to log-linear and Weibull distribution models. Similar to the oxygen plasma inactivation case, the two inactivation graphs were both of a log-linear shape, sharing very similar model parameters. The coefficients of determination values were both 0.99 and 0.99 for EGA and HPC, respectively. The results obtained by both methods were highly correlated with a correlation coefficient of 0.9998. EGA measured a *D*-value of 12.7 ± 0.7 min and *t*_*C*_ of 12.5 ± 1.0 min. HPC measured a *D*-value of 13.4 ± 0.8 min and *t*_*C*_ of 13.2 ± 1.1 min.

### Summary and Conclusions

When sterilization processes are evaluated using CFU inactivation of endospores (i.e., HPC), at least 2 days of incubation are required before results become available. Such a gap between sampling and results is undesirable for bioburden reduction or sterility assurance applications^58^. EGA provides the fastest possible biodosimetry, because it probes at the earliest stage of germination, and shows results on the timescale of minutes. We showed that EGA and HPC are reproducibly correlated in measuring endospores recovery efficiency from stainless steel coupons, and for measuring inactivation of endospores attached on stainless steel coupons with heat, ultraviolet radiation, vaporized hydrogen peroxide and oxygen plasma. EGA inherently measures a higher number of viable endospore than culture-based assays, because of the presence of germinable-but-not-culturable populations that are more resilient, which makes EGA a more conservative measure than HPC for sterility assurance. These populations arise because there are more mechanistic steps required in replication that can be targets for inactivation than those required for stage I germination. EGA is a fast, quantitative and complementary biodosimetry method to the traditional culture-based assays in sterility assurance testing of surfaces.

## Methods

### Chemicals

The methods used were described first in a Caltech thesis^59^. Terbium (III) chloride hexahydrate, 99.999%, L-alanine, DPA, and other salts were purchased from Sigma (St. Louis, MO) and were used as received. Agarose (>90%) was purchased from Invitrogen (Carlsbad, CA). Tryptic soy agar (TSA), nutrient broth and agar were obtained from Becton, Dickinson and Company (Sparks, MD). Calcium dipicolinate was prepared as previously reported^60^. Poly-dimethylsiloxane (PDMS) was obtained from Dow Corning (Edison, NJ).

### Endospore cultures

*Geobacillus stearothermophilus* ATCC 7953 was obtained in the form of filter paper inoculated with an endospore population of 1.8 × 10^6^ CFU per strip from Steris Corporation (Mentor, OH). *Bacillus subtilis* ATCC 27370 endospores were prepared as previously described^61^. *B*. *subtilis* was cultured in tryptic soy broth (TSB) at 37 °C for 36 h. Vegetative cells in the exponential growth phase were inoculated into a sporulation medium consisting of 1.6% nutrient broth, 1.5% agar, 0.2% KCl and 0.05% MgSO_4_ with filter-sterilized 1 mmol l^−1^ Ca(NO_3_)_2_, 100 µmol l^−1^ MnCl_2_, 1 µmol l^−1^ FeSO_4_, and 0.1% glucose. The sporulating culture was incubated at 37 °C for 5 days. Following an overnight lysozyme digestion at 37 °C, endospores were purified from vegetative cell and cell debris by repeated centrifugation at 15,000 x g for 15 min at 4 °C and washing until 99.9% fully refractile bodies was obtained under phase contrast microscopy.

*Bacillus atrophaeus* ATCC 9372 was obtained in endospore suspension from Raven Biological Laboratories, Inc. (Omaha, NE; now Mesalabs, Inc.), with a concentration of 2.3 × 10^10^ CFU ml^−1^. Total endospore concentrations were determined using a Petroff-Hausser hemocytometer (Horsham, PA) and CFU concentrations were determined using TSA HPC in triplicate measurements. Endospore suspension was stored at 4 °C in the dark before use. Before each germination experiment, endospore samples were centrifuged at 8000 × g for 20 min and washed twice to remove excess dipicolinic acid in the supernatant. No significant difference in the stock CFU population was observed before and after heat shock treatment at 80 °C for 15 min. The heat shock process is a standard protocol for evaluating the bioburden level of flight hardware for planetary protection in NASA’s robotic planetary missions as stated in the Handbook for the Microbial Examination of Space Hardware. The heat shock is used to kill vegetative cells, if present, and to activate the spores for germination.

### Coupon preparation

Stainless steel 316 sheets (40″ × 80″) were procured and cut into 2″ × 2″ coupons in the machine shop at the California Institute of Technology (Caltech). Cotton applicators (Puritan, Guilford, ME) of length 15 cm were used to swab the coupon surfaces. Coupons were rinsed with 18.2 MΩ-cm deionized water and acetone to remove surface impurities and stains. They were cleaned with clean-room grade polyester wipes (BD Consumer Healthcare, Franklin Lakes, NY) saturated with 70% isopropyl alcohol. Each coupon was autoclaved at 121 °C for 15 min inside a glass Petri dish before inoculation. Endospore suspensions of *B*. *atrophaeus* were added aseptically and evenly on the coupons. evaporation and deposition were observed under a microscope to ensure minimal clumping, even spread and formation of monolayers of endospores. Care was taken not to drip over the edges. Coupons were air dried at room temperature inside a biological safety cabinet for 2 h and then further dried inside a Petri dish in a desiccator for 12 h in the presence of silica gel, and were used within 24 h

### Surface sampling

Each coupon was swabbed three times using a moistened sterile cotton applicator at an angle of 30° applying a firm pressure on the surface. The same swabbing procedure was repeated two more times with a 90° and 45° rotation of the coupon, respectively. Double swabbing is used to minimize variations in pressure, sampling time and moistening of cotton tip (Patterson, 1971). The cotton head was aseptically broken off and placed into a tube containing 10 mL of sterile water. Cells were dislodged from the swab by 10 seconds of vortexing and 2 min of sonication at 25 kHz. The sample underwent heat shock at 80 °C for 15 min. Endospore strips of *G*. *stearothermophilus* and PDMS inoculated with *B*. *subtilis* were directly immersed into 10 mL of sterile water. The subsequent extraction and analysis processes were the same. A total of 30 samples at each inoculum population (0, 0.3, 1, 3.2, 10, 31.6, 100, 317, 1000 spore/coupon) were obtained on the stainless steel coupons. Germinable EGA counts were determined in replicates of 8 per coupon and culturable counts were determined in replicates of 6 per coupon, i.e., a total of 240 EGA measurements and 180 samples for culture assay.

### Enumeration of germinable endospores

Samples were filtered onto 1.5-mm^2^ spots on 0.2-µm polycarbonate membrane filters (Whatman, Florham Park, NJ) using a 96-well micro-sample filtration manifold (Schleicher and Schuell, Keene, NH). Endospores concentrated on the filter were transferred to a ~0.5 mm thick, 9 mm diameter slab of 1.5% agarose substrate containing 100 µmol l^−1^ TbCl_3_ and 20 mmol l^−1^ L-alanine mounted in a silicone isolator (Molecular Probes, Eugene, OR) on a quartz microscope slide. After transfer of endospores, the agarose surface was covered with a piece of 0.2-mm-thick PDMS (Dow Corning, Midland, MI), prepared by mixing the polymer base and curing agent in a 10 to 1 ratio by weight. After degassing, the mixture was casted over a 0.2-mm thick stainless steel mold and cured in an oven for 2 h at 65 °C. Agarose, silicone isolator and PDMS were autoclaved at 121 °C for 15 min before use. A piece of PDMS was peeled off and attached on top of an endospore-laden agarose surface aseptically for sealing. Time-lapse gated images were collected by real-time streaming with a delay of 100 µs and exposure time of 5 s in each frame using a previously reported EGA instrumentation^39^.

### Enumeration of culturable endospores

Culturable counts were determined by HPC on tryptic soy agar (TSA). *B*. *subtilis* and *B*. *atrophaeus* endospores were incubated at 37 °C. *G*. *stearothermophilus* endospores were incubated at 55 °C, CFU counts were recorded after 72 h of incubation.

### Heat inactivation

In the dry heat inactivation experiment, *B*. *atrophaeus* endospores inoculated on stainless steel coupons were heat treated in an oven at 105 ± 0.5 °C. A beaker of silica gel was kept inside the oven. The time required to raise the coupon surface temperature to 105 °C was determined by a thermocouple and was added to each exposure cycle. Lethality of the process only included the holding period at 105 °C. Immediately after removal from the oven, each stainless steel coupon was chilled by ice. Endospores were sampled by the swab-rinse technique and enumerated by TSA HPC and EGA. D_105 °C_ was determined from a best-fit regression line of the data points by using a least square method. The variation of the data points around each regression line was measured by calculating the standard deviation of the estimate.

### UV inactivation

*B*. *atrophaeus* endospores laden on stainless steel coupons were contained in glass Petri dishes. They were exposed to continuous irradiation of UV from a 4 W low-pressure mercury lamp (UVP, Upland, CA) with peak irradiance at 254 nm (UVC) coupled with a 0.7-neutral-density filter. The distance between the UV lamp and the surface of the stainless coupon was 10 cm. The intensity at 254 nm was 60 µW cm^−2^. The uniformity of the lamp source had a maximum variation of ~4% within the irradiation area. The lamp was switched on 20 min before the start of the inactivation to stabilize the emission power and spectrum. Irradiation experiments were performed in a dark environment at room temperature without controlling the relative humidity. After irradiation, the samples were immediately chilled by ice at 0 °C. The swab-rinse method was used to suspend sampled populations, and the endospore surface concentration was determined by EGA and CFU enumeration. The protocol for monitoring UV inactivation of *B*. *atrophaeus* endospores in suspension was previously reported^39^.

### Plasma inactivation

*B*. *subtilis* endospores were inoculated onto a 2 mm × 2 mm piece of PDMS placed on a glass microscope slide. The sample slide was placed within a glass vacuum chamber (150 mm radius and 300 mm length) at room temperature of a plasma system (Diener Electronics GmbH + Co., Germany). Low pressure was achieved using a vacuum pump. A radio frequency of 13.6 MHz was used to sustain the plasma. The endospore-inoculated glass slides were exposed to glow discharge at 100 W power for 0, 15, 30, 45, and 60 min. The RF generator was turned off immediately at the end of the inactivation process.

### Vaporized hydrogen peroxide inactivation

Vaporized hydrogen peroxide (VHP) resistance *of G*. *stearothermophilus* ATCC 7953 (mean population = 1.8 × 10^6^ CFU/coupon) on metal coupons was measured by EGA and culturability (colony formation on TSA). In a preliminary test, 0.5 mL of hydrogen peroxide was vaporized at 45 °C for 3 min in a chamber at Jet Propulsion Laboratory. 10^7^ *B*. *atrophaeus* endospore/coupon and 1.8 × 10^6^ *G*. *stearothermophilus* endospore/coupon were placed inside sterilization pouches and subjected to vaporized H_2_O_2_ inactivation. In the VHP experiment, *G*. *stearothermophilus* endospores strips with different durations of vaporized H_2_O_2_ inactivation treatment (0, 15, 30, 45, 60 min) were obtained from Steris Corporation using a LaCalhene two-glove isolator connected to a VHP1000ED system. *G*. *stearothermophilus* endospores were incubated and germinated at 37 °C and 55 °C, respectively.

### Data analysis

Phase contrast microscopy (PCM), Endospore Germinability Assay (EGA), and heterotrophic plate counts (HPC) were used to determine total endospore populations (P_PCM_), germinable populations (P_EGA_) and culturable populations (P_HPC_), respectively, and these were used to derive the germinable fractions (P_EGA_/P_PCM_) and culturable fractions (P_CFU_/P_PCM_). Average percent recovery (%R) of endospores is calculated as follows:

$$ \% {R}_{{\rm{EGA}}}=\frac{{P}_{{\rm{EGA}}({\rm{recovered}})}}{{P}_{{\rm{EGA}}({\rm{despoited}})}}\times 100 \% $$

$$ \% {R}_{{\rm{HPC}}}=\frac{{P}_{{\rm{HPC}}({\rm{recovered}})}}{{P}_{{\rm{HPC}}({\rm{despoited}})}}\times 100 \% $$

Germinable and culturable populations were analyzed by using a logarithmic transformation. Average of the results were compared using the Mann-Whitney *U*-test. The correlation between endospore counts was analyzed by using a simple linear regression analysis and Spearman’s rank correlation coefficient. A square root transformation was employed on the analysis of variance in the low concentration inoculum regime (<100 spores/coupon), where the endospore count followed a Poisson distribution. All data were analyzed at the 95% confidence level. Differences between mean values were calculated by Fisher’s least significant difference (LSD) values, when appropriate. Differences were considered significant if *p* < 0.05.

### Inactivation models

Endospore survival graphs were constructed by plotting endospore numbers in logarithm versus inactivation times. The survival graphs were fitted to both Bigelow first-order model and Weibull distribution model. The Bigelow first-order model is given by:

$$log(\frac{P}{{P}_{0}})=-\,\frac{t}{D},$$

where *P*_0_ is the initial number of endospores and *P* is the number of endospores at time *t* measured immediately after the inactivation. Endospore inactivation results are reported in terms of both P_EGA_, and P_CFU_. Decimal reduction values (*D*-values) were calculated based on the log-linear portion of the plot. Endospore survival graphs were also fitted to the Weibull distribution model to better describe nonlinear features such as concavity, shoulders and tails^39^, which is given by:

$$log(\frac{P}{{P}_{0}})={e}^{-{(t/\alpha )}^{\beta }}$$

where *α* is the scale parameter in time, and *β* is the dimensionless shape parameter. In a semi-logarithmic plot, the Weibull distribution corresponds to a concave upward survivor curve when *β* > 1, concave downward curve if *β* < 1, and is linear if *β* = 1. A parameter, *t*_*C*_, can be used to represent resistance or susceptibility towards certain inactivating agents, similar to the use of *D*-values in log-linear model.

$${t}_{C}=\alpha \cdot \Gamma (1+{\beta }^{-1})$$

where Γ is the gamma function. Goodness-of-fit was assessed using regression coefficient (*R*^2^).

## Data Availability

The datasets used and analysed during the current study are available from the corresponding author on request.
